# Stratified association of Hippocampus volume with normal serum Sodium levels and predictive analyses of cognitive adversities in non-demented middle-aged and older adults

**DOI:** 10.1192/j.eurpsy.2026.10732

**Published:** 2026-07-31

**Authors:** A. Hallab

**Affiliations:** Charité Universitätsmedizin Berlin, Berlin, Germany

## Abstract

**Introduction:**

Serum Sodium abnormalities are largely observed in older adults and are associated with higher risks. Less is known about the association between normal serum Sodium variations and medial temporal brain structures, mainly involved in cognition and memory.

**Objectives:**

The study’s objective was to explore the association between serum Sodium and Hippocampus volume and to assess associated cognitive risks.

**Methods:**

Non-demented ADNI3 participants (healthy controls (HC) and those with mild cognitive impairment (MCI)) with complete serum Sodium, ADAS_13_ score, and Hippocampus volume at baseline were included. Linear and non-linear associations were evaluated. To assess the odds of MCI, logistic regression was performed.

**Results:**

A total of 469 cases with a median age of 70 years (IQR: 66, 76) were included. The median serum Sodium level was 141 (IQR: 139, 142). Serum Sodium levels showed a significant association with Hippocampus volume in the MCI subgroup (Adj.*ß*_MCI_=-95 (-162, -28), *p*=0.006, *q*=0.036).

Serum Sodium levels did not show a significant association with the ADAS13 total score (Adj.*ß*_Total_=-0.04(-0.28, 0.21), *p*=0.8) or with the odds of being diagnosed with MCI at baseline (OR_MCI_= 1.00(0.88, 1.13), *p*= 0.935).

**Conclusions:**

Normal serum sodium variations were significantly associated with hippocampus volumes, depending on the underlying neurodegenerative pathology, without predicting clinically relevant cognitive decline. Further studies are needed to better understand the mechanisms and identify protective factors.

**Disclosure of Interest:**

None Declared

